# Effects of Cd treatment on morphology, chlorophyll content and antioxidant enzyme activity of *Elymus nutans* Griseb., a native plant in Qinghai-Tibet Plateau

**DOI:** 10.1080/15592324.2023.2187561

**Published:** 2023-03-20

**Authors:** Ying Hu, Huichun Wang, Huiping Jia, Maodeji Peng, Tiantian Zhu, Yangyang Liu, Jingjing Wei

**Affiliations:** a College of Life Sciences, Qinghai Normal University, Xi’ Ning, China; b Qinghai south of Qilian Mountain Forest Ecosystem Observation and Research Station, Huzhu; c Key Laboratory of Medicinal Animal and Plant Resources on the Qinghai–Tibet Plateau, Qinghai Normal University, Xi’ Ning, China; d College of Geographical Sciences, Qinghai Normal University, Xi’ Ning, China

**Keywords:** *Elymus nutans* Griseb., cadmium treatmentes, growth, malondialdehyde, antioxidant enzyme, photosynthetic pigment

## Abstract

Cd pollution is a global environmental problem. However, the response mechanism of the alpine plant Pelagia under Cd stress remains unclear. Therefore, in this study, a native plant(*Elymus nutans* Griseb.) of the Qinghai-Tibet Plateau was used as the material to quantify plant height, leaf number, length of leaf, crown width, root number, biomass, Dry weight malondialdehyde (MDA), free proline, superoxide dismutase (SOD), ascorbate enzyme (APX), catalase (CAT) and chlorophyll contents under different Cd concentrations. The results showed that the growth of *Elymus nutans* Griseb. was a phenomenon of “low concentration promotes growth, high concentration inhibited growth” under Cd treatment. It meant that 10 mg·L^−1^ Cd promoted the growth of leaf number, plant height, crown width and tiller number, while 40 mg·L^−1^ Cd inhibited the growth of root number and biomass of *Elymus nutans* Griseb. compare with the control. The MDA content, free proline content, SOD activity, APX activity and CAT activity of *Elymus nutans* Griseb. was increased with the increase of Cd treatment concentration to resist the oxidative damage caused by Cd to the body. At the same time, the accumulation of chlorophyll A, chlorophyll B and chlorophyll AB was decreased with the increase of Cd stress concentration. In addition, the carotenoid content did not change much between the control group and the treatment group, indicating that Cd treatment had little effect on it. The results could provide a reference for the mechanism of heavy metal resistance and the selection and improvement of Cd -resistant varieties of *Elymus nutans* Griseb.

## 1. Introduction

Heavy metal pollution is mainly caused by human activities and natural factors, among which Cd is a highly toxic environmental pollutant, which is difficult to degrade and easy to move in soil^1,2^. In the process of plant growth, Cd is a non-essential element that enters the plant body after being absorbed by plant roots, and has toxic effects on plant roots, stems, and leaves, such as chlorosis, necrosis, and growth inhibition^3^. At the same time, Cd enters the food chain and food web through, and accumulates in other organisms, eventually causing harm to the organisms of the entire ecosystem, and human health is also greatly threatened^4^. At present, humans use three methods of chemical, physical and biological to repair the environment polluted by heavy metals. Among them, chemical and physical repair have problems, such as high cost, new pollutants introduced, and difficulty in implementing large areas^5^. On the contrary, bioremediation is an environmentally friendly method, which has the advantages of low cost, no secondary pollution, and large area use. It is a heavy metal pollution remediation method that is worthy of in-depth study and utilization. It has become hot spots of heavy metal pollution remediation technology^6^. Phytoremediation technology is an important component of bioremediation technology. At present, there have got a large number of reports on the basic research of plant absorption of Cd. For example, soil Cd has negative consequences for the growth of sassafras. It reduces the net growth of planting height and leaves, branches and roots^7^. Cd treatment holds different degrees of inhibition on the radicle length of different varieties of maize, and the maximum degree of inhibition can reach 88.89% of the radicle length of the control^8^. In addition, Cd can also have a huge impact on plant physiology, such as reducing respiratory intensity and photosynthetic rate, and the biological toxic process of protein degradation^9^. Meanwhile, it can also cause high lipid peroxidation and change the activity of antioxidant enzymes^10^, etc. In Dendrobium officinale seedlings, 14 mg·L^−1^ Cd treatment significantly increased antioxidant levels and induced the accumulation of malondialdehyde and proline. In addition, metal transporter, sulfate glutathione metabolism, cell wall metabolism and phenylpropanine metabolism played an important role in Cd stress adaptation^11^. Under Cd stress, the photosynthetic rate and pigment levels, the activities of ascorbate peroxidase (APX), guaiacol peroxidase (GPX), catalase (CAT) and superoxide dismutase (SOD) were inhibited, and the level of malondialdehyde (MDA) was increased of Eruca sativa Mill^12^. For sugar beet (*Beta vulgaris* L.), 0.5 and 1 mM Cd inhibited the plant height, shoot and root growth of beet seedlings to varying degrees. The leaves gradually withered and turned yellow, and the roots turned dark brown. With the increase of Cd concentration, the content of malondialdehyde and the activities of peroxidase, superoxide dismutase and glutathione S-transferase were increased to varying degrees^13^.

The Qinghai-Tibet Plateau is about 2 500 kilometers long from east to west, and about 1,000 kilometers wide from north to south. It occupies about 2% of the Earth’s land surface. It is under a high altitude and a cold climate (26°N-40°N). It is called the “Asian Water Tower” due to its large amount of dense water and “Third Pole”^14^. Studies have demonstrated that heavy metal pollution such as Cr, Cu, Cd, Pb, Zn has gradually appeared in the northeastern part of the Qinghai-Tibet Plateau^15^. This area has become a high-risk area for cancer, and industry is the primary source of pollution^16^. However, there are few reports on Cd pollution remediation by native plants on the Qinghai-Tibet Plateau. Therefore, this study selected the Qinghai-Tibet plateau native plants with *Elymus nutans* Griseb.(*En*) as the research material, research it under the biological toxicity and root length, plant height and biomass growth and free proline, malondialdehyde, antioxidant enzymes and the change of physiological indexes such as chlorophyll of leaf of Cd treatment, in order to provide a certain reference phytoremediation of Cd pollution of the Qinghai-tibet plateau.

## 2. Materials and Methods

### 2.1 Glasshouse culture

The cultivated *Elymus nutans* Griseb.(*En*) seeds were provided by Tongde Ranch in Qinghai Province, China (35°15′N, 100°38′E, Xiaolong Quan et al. 2021). The mature and full eldrine seeds were selected, soaked in distilled water for 12 hours. The disinfect with 10% H_2_O_2_ for 30 min, and finally rinse with distilled water for at least three times, and use filter paper to accommodate the excess water on the surface of the seed for use^17^. Sow treated seed in a 90 mm diameter pewter dishes. Before sowing the seed, the petri dish is spread with about 0.2 cm diameter vermiculite and soaked with distilled water. The petri dishes seeded with En seeds were carefully cultured in an AI climate incubator. The cultural condition is 14 h light (intensity 5000 lX) 25 ± 2°C humidity 80%, 10 h dark treatment, 20 ± 2°C growth.

**Figure 1.**
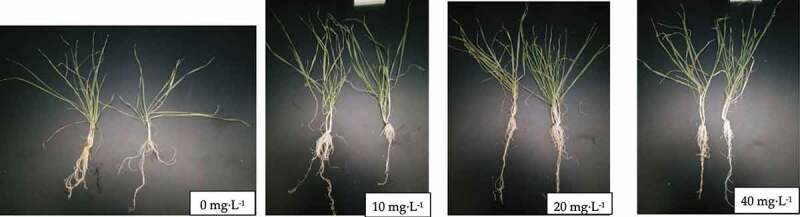
Plant graphs of different concentration gradients after cadmium treatment.

We transplanted the cultivated *En*. after 15 days of cultivation, select *En* condenses seedlings with basically the seedlings into a cylindrical glass culture tank (the size of the culture tank is 10 cm high and 5 cm in diameter), and the treatment test was carried out after 7 days of cultivation with Hoagland nutrient solution. This helped the transplanted *En* seedlings adapt to the new environment. The stress test set up four treatments, Cd concentration gradient of 0, 10, 20, 40 mg·L^−118^, Cd reagent in the form of CdCl_2_·2.5 H_2_O, and the stress solution used in the test was configured with Hoagland nutrient solution, in order to ensure sufficient nutrients for plant growth. There were 3 replicates in each treatment, and 20 seedlings were transplanted in each replicate. After the stress solution was added for the first time, Cd free plant nutrient solution was added regularly to ensure the normal growth of plants. The simulated outdoor growth conditions were 25 ± 2°C and 14 h illumination in the greenhouse during the day. At night, the greenhouse was 20 ± 2°C and dark for 10 h. After 28 days of stress culture, the plants were harvested. The plant picture is shown in Figure 1.

### 2.2 Assay method

We measured plant height, leaf length and crown width with a ruler, and recorded the number of leaves and tillers at the same time. The measurement time cycle was measured once every 2 days. After harvest, root length and plants hight were measured with a tape measure, and Electronic analytical balance is a tool for measuring biomass. Plant materials were stored in a refrigerator at −80°C^19^. Referring to previous studies, plant physiological indexes were determined^20–22^. The malondialdehyde (MDA) content was measured by thiobarbituric acid colorimetric method, and the activity of superoxide dismutase (SOD) of antioxidant enzyme system was measured by NBT photoreduction colorimetric method, SOD activity unit is 50% of the inhibition of NBT photochemical reduction as one enzyme activity unit (U). Catalase (CAT) and ascorbate catalase (APX) activities were detected by UV spectrophotometry, CAT and APX enzyme activities were reduced by 0.01 OD value per minute for 1 enzyme activity unit (U). Free proline was determined by acid ninhydrin colorimetry. The chlorophyll of plant leaves was extracted by acetone. The absorption values at 663 nm, 645 nm and 652 nm were determined, and the contents of chlorophyll A, chlorophyll B, chlorophyll AB and carotene were calculated.

### 2.3 Statistical method

Data were statistically analyzed using Statistical Product Service Solutions(SPSS) 17.0 (SPSS Inc., Chicago, USA) version software. One-way analysis of variance (ANOVA) was used to determine the variability and validity of the results, and Duncan’s Multiple Range test (DMRT) was used to determine the significance between treatments. Correlation analysis was carried out on all data about Cd content changes or parameters, and normality test was performed before data processing in each group marked in the corresponding position, where * means *p* < 0.05, ** means *p* < 0.01, *** means *p* < 0.001^23^.

## 3. Results

### 3.1 Effects of Cd treatment on the growth of En

**Figure 2.**
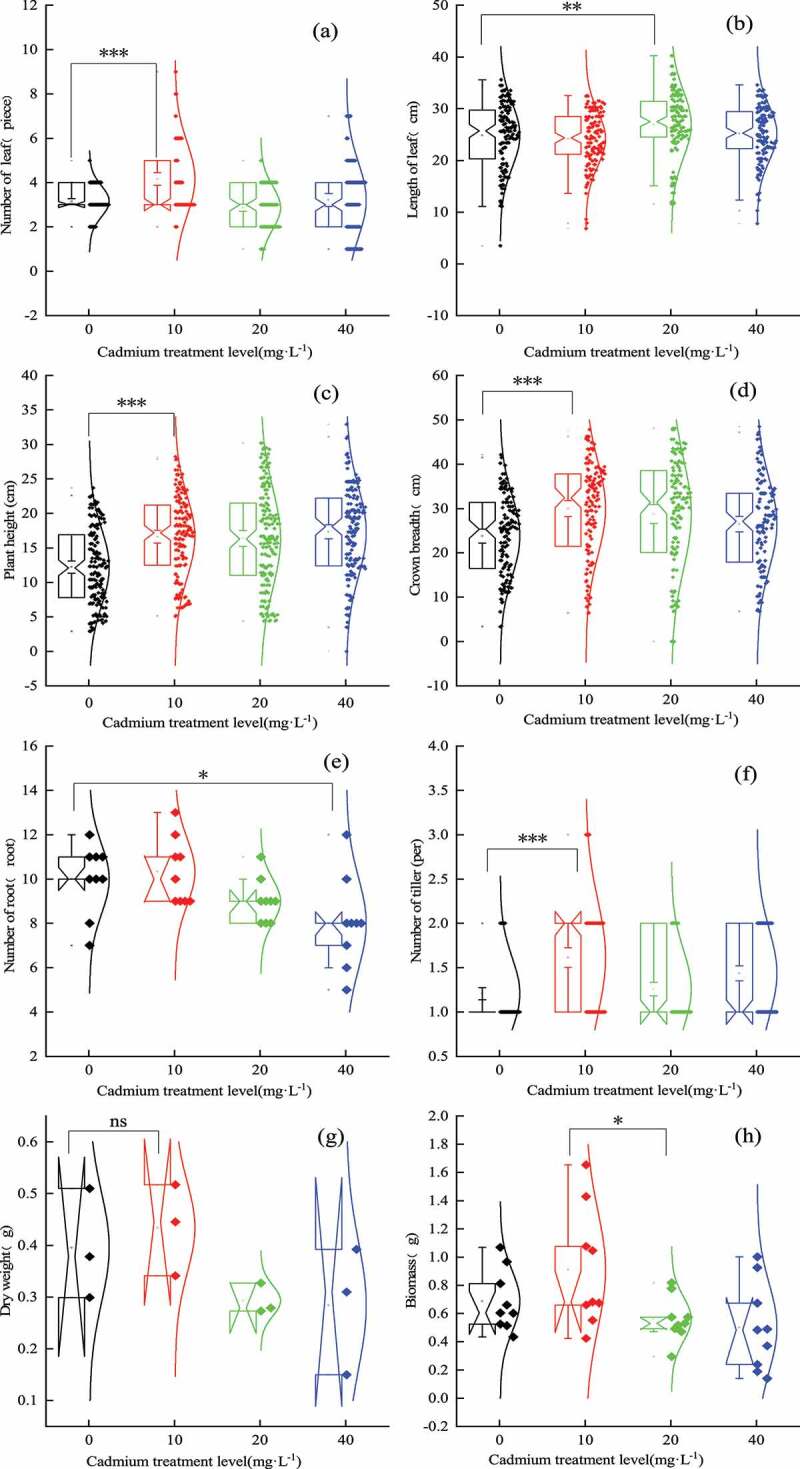
Effects of Cd treatment on the growth. (a) represents the number of leaves, F=2.347***; (b) represents leaf length, F=5.283***; (c) plant height, F=9.891***; (d) for crown width, F=25.924***; (e) the case that represents the number of roots, F=2.971***; (f) represents tiller number, f =3.838***; (g) represents dry weight, F=3.445***; (h) represents biomass, F=3.445***.

With the increase of Cd treatment concentration, the number of *En* leaf first increased and then decreased, and was the highest in 10 mg·L^−1^ treatment group (Figure 2a, *p <* 0.001). Leaf length also increased first and then decreased, and was the highest in the 20 mg·L^−1^ treatment group (Figure 2b, *p <* 0.001). In terms of plant height growth, the plant height of all treatment groups was basically the same and higher than that of the control group (Figure 2c, *p <* 0.001). The change in crown width of *En* was the same as the change in the number of leaves. The 10 mg·L^−1^ treatment group reached the highest (Figure 2d, *p <* 0.001), and the number of roots also reached the highest in the 10 mg·L^−1^ treatment group (Figure 2e, *p <* 0.001). The number of tillers fluctuates to a certain extent, which first increases and then decreases and then increases. Among them, the 10 mg·L^−1^ treatment group reached the highest (Figure 2f, *p <* 0.001). In addition, the dry weight of *En* showed no difference between the control group and the treatment group (Figure 2g, *p <* 0.001), while the dry weight of it increased first and then decreased with the increase of Cd treatment concentration, and reached the highest in the 10 mg·L^−1^ treatment group, which had a significant promoting effect (Figure 2h, *p <* 0.001). In general, 10 mg·L^−1^ Cd treatment promoted the growth of leaf number, plant height, crown width and tiller number, while 40 mg·L^−1^ Cd treatment inhibited the growth of root number and biomass of *En*.

### 3.2 Effects of Cd treatment on physiology of En

**Figure 3.**
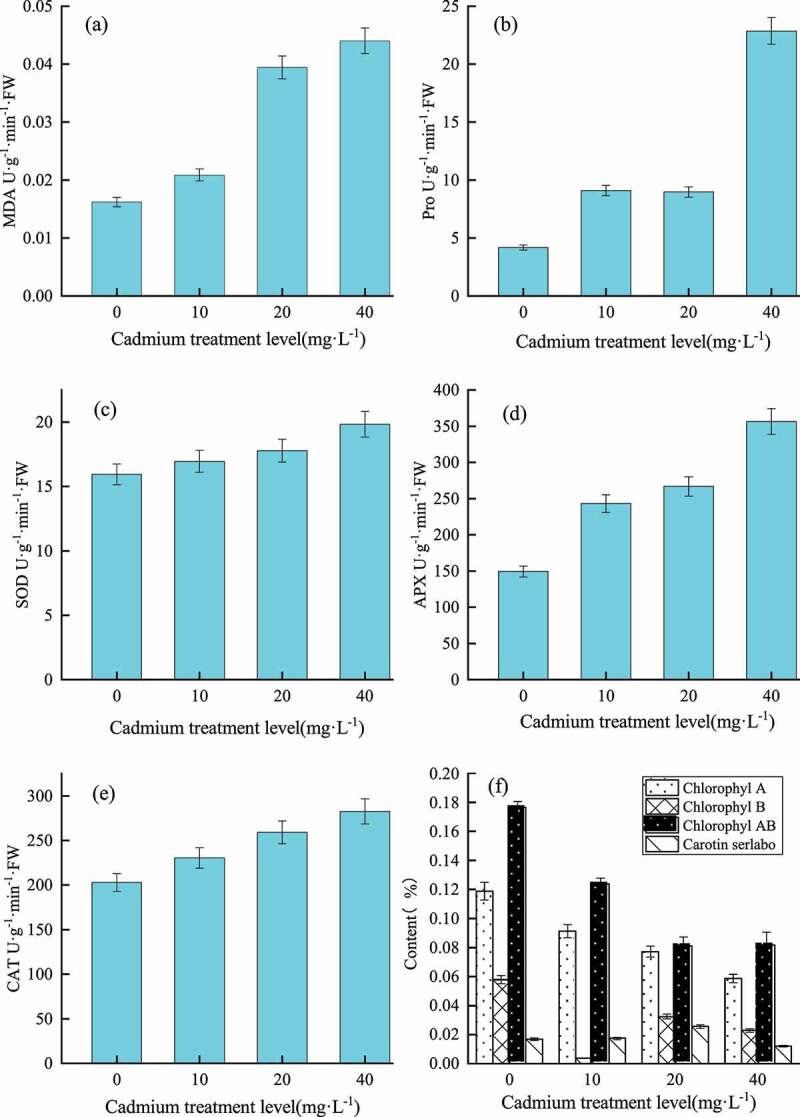
Effects of Cd treatment on physiology of *En*. (a) represents the change of MDA, F=734.298***; (b) represents the change of free proline, F=940.261***; (c) SOD change, *t*=21.346***; (d) represents APX change, *t*=5.969***; (e) represents CAT change, *t*=11.697**; (f) represents the change of chlorophyll, wherein, chlorophyll A, F=3998.610***, chlorophyll B, F=2935.059***, chlorophyll AB, F=7653.933***, carotene, F=305.473***.

With the increase of Cd treatment concentration, MDA content (Figure 3a, *p <* 0.001), free proline content (Figure 3b, *p <* 0.001), SOD activity (Figure 3c, *p <* 0.001), APX activity (Figure 3d, *p <* 0.001) and CAT activity (Figure 3e, *p <* 0.001) all showed an increasing trend. At the same time, under Cd treatment, the contents of chlorophyll A(*p<*0.001), chlorophyll B (*p<*0.001) and chlorophyll AB (*p<*0.001) in *En* leaves decreased with the increase of Cd stress concentration, indicating that Cd stress inhibits chlorophyll synthesis and photosynthesis damage in it. The carotenoid content of all treatment groups was basically similar, indicating that Cd treatment had little effect on them (Figure 3f, *p <* 0.001).

### 3.3. Cluster analysis of growth and physiology of En treated by Cd

**Figure 4.**
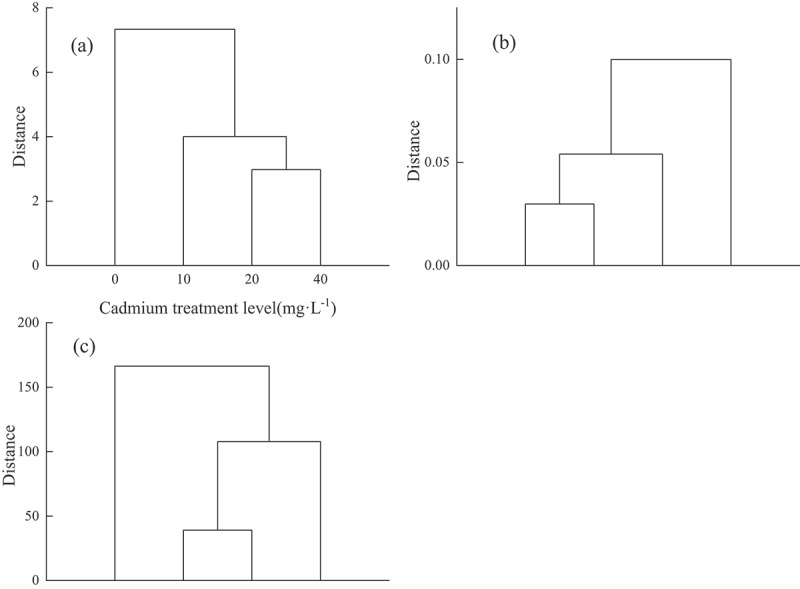
Cluster analysis of Cd treatment on En growth and physiology. (a) clustering analysis of growth indicators; (b) clustering analysis of chlorophyll; (c) the clustering analysis of MDA, free proline, SOD, APX and CAT.

We used systematic cluster analysis to analyze the growth index, chlorophyll content, MDA, free proline, SOD, APX and CAT of *Elymus elymus* under Cd treatment. This study showed that different concentrations of heavy metals treated *Elymus nutans* Griseb. Although they can be clustered into three categories, the growth indexes were clustered at 0, 10, 20–40 mg·L^−1^, while the chlorophyll content and antioxidant enzyme activity were clustered at 0–10, 20, 40 mg·L^−1^ and 0, 10–20, 40 mg·L^−1^, respectively. It indicating that they are sensitive to different concentrations of Cd (Figure 4).

## 4. Discussion

The growth index can reflect the Cd toxicity of plants at the individual level, while the physiological index can judge the effects of Cd toxicity at the molecular level of plants and reveal the response of plants to such external stimuli to a certain extent. In this paper, the growth and development response of *En* under Cd treatment was studied. Compared with control group, 10 mg·L^−1^ Cd treatment promoted the growth of it leaf number, plant height, crown width and tiller number, while 40 mg·L^−1^ Cd treatment inhibited the growth of it root number and biomass. This was consistent with the research results that Cd treatment “low concentration promotes growth, high concentration inhibits growth” affects plant growth^24^. In addition, after the first addition of Cd stress solution, we used Cd-free Hoagland nutrient solution, which may have diluted the Cd concentration in the stressed environment, so that the plant morphological growth was not inhibited, but promoted. In a follow-up study, we found that the morphological growth of plants would be significantly inhibited if Cd-containing nutrient solution was continuously added. This condition may be related to plant growth regulators, which mainly include ababic acid (ABA), auxin, brassin steroid (BR), cytokinin (CK), ethylene (ET), gibberellinic acid (GA3), jasmonic acid (JA), salicylic acid (SA), nitric oxide (NO) and polyamine (PAs)^25^. Under high cadmium treatment, ABA concentration in plants increases, which can control root tip cell proliferation to limit taproot development. In addition, the combination of ABA and auxin mediates seed dormancy, which is required for seed dormancy, while ABA inhibits seed germination. Plants achieve germination and dormancy by regulating the ratio of the two substances in stressed environments^26,27^. ABA has antagonistic effect with gibberellin (GAs), and is involved in regulating seed germination, seed dormancy, plant growth, flowering and fruit ripening^28^. Salicylic acid (SA) plays an important role in plant development. Spraying SA significantly reduced the accumulation of Cd in rice grains and promoted the deposition and fixation of Cd on the cell wall of leaves, thus reducing the transfer of Cd from leaves to grains during the filling stage. At the same time, SA reduced the accumulation of H_2_0_2_ and MDA in rice leaves and increased the chlorophyll content, thus reducing the toxicity to Cd. In addition, spraying SA reduced the inhibitory effect of Cd on plant height of rice seedlings during vegetative growth period, increased the dry weight of seedlings and reduced the transfer of Cd from leaves to grains, thus reducing the Cd content in rice^29^. Jasmonic acid increased the cell wall regionalization of Cd and inhibited the movement of Cd to protoplasts by promoting the binding of Cd to the root complex soluble pectin of rice (*Oryza sativa* L.), thus reducing the Cd content in roots and shoots by 30.5% and 53.3%, respectively. Jasmonic acid can reduce the H_2_O_2_ content in leaves by increasing the levels of catalase (CAT), peroxidase (POD), ascorbate peroxidase (APX) and glutathione (GSH), and reduce the damage of membrane lipid peroxidation induced by Cd, but has no significant effect on the activity of superoxide dismutase (SOD)^30^. Cluster analyses are unsupervised machine-learning algorithms that aim to delineate subgroups in datasets, characterized by discrete differences^31^. In this paper, cluster analysis showed that the morphological indexes, antioxidant enzyme activity and chlorophyll content of *En* were different under cadmium stress, and their change patterns were inconsistent, which may be caused by the metabolic pathway of heavy metal cadmium in plants. Generally, heavy metal first reaches the plant cell wall after being absorbed by plant roots^32^, and then crosses the cell membrane to reach the cytoplasm. The heavy metals are then bound by chelates in the plant cytoplasm (such as amides, malic acid and other organic acids) to form nontoxic complexes, which are then transported to vacuoles for storage^33^. Proline, glutathione, protein and plant hormones are closely related to plant resistance to heavy metals, and are involved in regulating the physiological phenomenon of abnormal increase of plant ROS^33–35^. To prevent heavy metal-induced Ros damage, plants rely primarily on metabolically mediated natural compounds such as phytochelin (PCs), reduced glutathione (GSH), carotenoids, and tocopherols, as well as enzymatic antioxidant systems, these include catalase (CAT), superoxide dismutase (SOD), ascorbate peroxidase (APX), peroxidase (POD), guaiacol peroxidase (GPX), etc. Increased levels of these metabolic intermediate compounds and antioxidant enzymes lead to increased stress tolerance to heavy metal induced Ros^36–38^. MDA is an indicator of lipid membrane peroxidation intensity and damage degree of membrane system, and the larger the value is, the more serious the damage^24^. The results of this study show that compared with the control group, MDA content in the treated group increased with the increase of treatment concentration, indicating that in the process of the increase of external Cd concentration, the peroxidation intensity of eldrine lipid membrane and damage degree of membrane system increased continuously, causing oxidative damage^39^. This was similar to the results of Zhang xiong Han et al., MDA content of Rape (*Brassica Napus* L.) increased under 0.5 and 6 mg/kg Cd treatment^40^. The hydrophobic end of free proline can bind protein and the hydrophilic end can bind water molecules to help protein bind more water molecules and prevent protein from dehydration and denaturement under treatment. In case of heavy metal treatment, the content of free proline increases to protect cell protein from damage^9^. The content of free proline in *En* increased with the increase of Cd treatment concentration, indicating that the increase of free proline synthesis in *En* is one of its Cd detoxification mechanisms^41^. This was consistent with the results reported by Ertan et al that Cd stress increases free proline in Rocket (*Eruca sativa* L.)^42^. SOD, APX and CAT are the main components of antioxidant enzymes, which protect plants from oxidative damage by removing excess reactive oxygen species in plants^43^. These enzymes play a crucial role in balancing the production and breakdown of Ros in the organism, and they work collaboratively to remove Ros, as no single enzyme can remove all forms of ROS alone^44^. The superoxide anion (O_2_-·) is reduced by SOD to form molecular oxygen and hydrogen peroxide (H_2_O_2_), which is then reduced to H_2_O by CAT, or oxidized by GPx to form H_2_O by two GSH molecules to form glutathione disulfide (GssG), which is reduced by GR at NADPH consumption. GST cleavage glutathione binding to other biomolecules^45^. In this study, it was found that the SOD, APX and CAT enzyme activities in *En* increased with the increase of Cd treatment concentration. This was consistent with the results of Fanwei Dai et al., who reported a significant increase in antioxidant enzyme activity of *Morus atropurpurea* under Cd stress compared to the control group^46^. Meanwhile, Yang et al. also found that Cd stress seriously affected the growth of *Elymus dahuricus*, induced the production of reactive oxygen species in clover leaves and roots, led to membrane lipid peroxidation, and activated the antioxidant defense system^47^. Chlorophyll is an important substance for plants to connect the inorganic environment with their own organic life, and is also a key substance to ensure the energy source of all heterotrophic organisms. The results showed that the contents of chlorophyll A, chlorophyll B and chlorophyll AB decreased with the increase of Cd stress, indicating that Cd stress could cause photosynthesis damage in *En*^48^. This was consistent with the results of Ying et al.‘s study that Cd treatment reduces the chlorophyll content of two Taxodium clones^49^. Even for hardy invasive grasses, Cd exposure can cause photosynthetic damage^50^. In addition, carotene in every little change between treatment group of Cd, it may be less affected by Cd treatment.

## 5. Conclusions

In this study, *Elymus nutans* Griseb, an excellent alpine forage with tolerance to cold, cold and salt and alkali, was used. As experimental materials, to explore its morphological growth, chlorophyll content and antioxidant enzyme activity under different cadmium concentrations, and to provide new insights for the species selection of cadmium pollution remediation in alpine regions (mines, farmland and urban landfills, etc.). At the same time, it also provides reference for the cadmium stress mechanism of plants growing in cold and high altitude climate environment. In the results of our study, under heavy metal treatment, 10 mg·L^−1^ treatment can promote the growth of *En* leaf number, plant height, crown width and tiller number. In addition, *En* could resist the damage caused by Cd by increasing the content of MDA, free proline and antioxidant enzyme. Contrary to the changes of antioxidant enzymes, Cd stress reduced the chlorophyll content of *En* and caused photosynthetic damage.

## Data Availability

The datasets generated during and/or analyzed during the current study are available from the corresponding author on reasonable request https://orcid.org/0000-0002-1274-8943.
